# Lipoprotein(a) Particle Production as a Determinant of Plasma Lipoprotein(a) Concentration Across Varying Apolipoprotein(a) Isoform Sizes and Background Cholesterol‐Lowering Therapy

**DOI:** 10.1161/JAHA.118.011781

**Published:** 2019-03-22

**Authors:** Dick C. Chan, Gerald F. Watts, Blai Coll, Scott M. Wasserman, Santica M. Marcovina, P. Hugh R. Barrett

**Affiliations:** ^1^ School of Medicine University of Western Australia Perth Australia; ^2^ School of Biomedical Science University of Western Australia Perth Australia; ^3^ The Lipid Disorders Clinic Department of Cardiology Royal Perth Hospital Perth Australia; ^4^ Amgen Inc. Thousand Oaks CA; ^5^ Northwest Lipid Metabolism and Diabetes Research Laboratories Division of Metabolism, Endocrinology, and Nutrition Department of Medicine University of Washington Seattle WA; ^6^ Faculty of Medicine and Health University of New England Armidale New South Wales Australia

**Keywords:** apolipoprotein, cardiovascular disease risk factors, cholesterol‐lowering drugs, lipids and lipoprotein metabolism, low‐density lipoprotein, Lipids and Cholesterol, Metabolism, Pathophysiology

## Abstract

**Background:**

Elevated lipoprotein(a) (Lp(a)), a low‐density lipoprotein‐like particle bound to the polymorphic apolipoprotein(a) (apo(a)), may be causal for cardiovascular disease. However, the metabolism of Lp(a) in humans is poorly understood.

**Methods and Results:**

We investigated the kinetics of Lp(a)‐apo(a) and low‐density lipoprotein‐apoB‐100 in 63 normolipidemic men. The fractional catabolic rate (FCR) and production rate PR) were studied. Plasma apo(a) concentration was significantly and inversely associated with apo(a) isoform size (*r*=−0.536, *P*<0.001) and apo(a) FCR (*r*=−0.363, *P*<0.01), and positively with apo(a) PR (*r*=0.877, *P*<0.001). There were no significant associations between the FCRs of apo(a) and low‐density lipoprotein‐apoB‐100. Subjects with smaller apo(a) isoform sizes (≤22 kringle IV repeats) had significantly higher apo(a) PR (*P*<0.05) and lower apo(a) FCR (*P*<0.01) than those with larger sizes. Plasma apo(a) concentration was significantly associated with apo(a) PR (*r*=0.930, *P*<0.001), but not with FCR (*r*=−0.012, *P*>0.05) in subjects with smaller apo(a) isoform size. In contrast, both apo(a) PR and FCR were significantly associated with plasma apo(a) concentrations (*r*=0.744 and −0.389, respectively, *P*<0.05) in subjects with larger isoforms. In multiple regression analysis, apo(a) PR and apo(a) isoform size were significant predictors of plasma apo(a) concentration independent of low‐density lipoprotein‐apoB‐100 FCR and background therapy with atorvastatin and evolocumab.

**Conclusions:**

In normolipidemic men, the plasma Lp(a) concentration is predominantly determined by the rate of production of Lp(a) particles, irrespective of apo(a) isoform size and background therapy with a statin and a proprotein convertase subtilisin‐kexin type 9 inhibitor. Our findings underscore the importance of therapeutic targeting of the hepatic synthesis and secretion of Lp(a) particles. Lp(a) particle catabolism may only play a modest role in determining Lp(a) concentration in subjects with larger apo(a) isoform size.

**Clinical Trial Registration:**

URL: http://www.clinicaltrials.gov. Unique identifier: NCT02189837.

**Clinical Perspective**

**What Is New?**

- The present study shows that plasma lipoprotein(a) (Lp(a)) concentration is predominantly determined by the rate of production of Lp(a) particles independent of apolipoprotein(a) isoform size and treatment with atorvastatin and evolocumab.
- Lp(a) particle catabolism only plays a modest role in determining Lp(a) concentration in subjects with larger apolipoprotein(a) isoform size.

**What Are the Clinical Implications?**

- The present study advances knowledge of the in vivo physiology of Lp(a) particles, suggesting that therapies for lowering plasma Lp(a) concentration should target the hepatic synthesis and secretion of apolipoprotein (a).

## Introduction

Classical and Mendelian epidemiological studies demonstrate that elevated plasma concentrations of lipoprotein(a) (Lp(a)) may be related to the development of atherosclerotic cardiovascular disease (ASCVD).^1^, ^2^, ^3^, ^4^, ^5^ The pathobiology appears to entail the infiltration of Lp(a) into the subendothelial space, where it may promote foam cell formation, oxidative stress, inflammation, and thrombosis.^2^ However, the metabolism of Lp(a) in humans remains enigmatic and requires clarification to enhance the clinical understanding of this major heritable risk factor.

Lp(a) is a polymorphic lipoprotein synthesized by the liver and comprises 1 molecule of the glycoprotein apolipoprotein(a) (apo(a)) covalently bound to apoB‐100‐containing low‐density lipoprotein (LDL)‐like particles.^5^ We recently demonstrated that the transport of apo(a) and apoB‐100 proteins within Lp(a) particles is tightly coupled in the circulation.^6^ The plasma concentrations of Lp(a) have a strong heritable component related to genetic variations in the apo(a) moiety, which in some studies have been independently associated with the incidence of ASCVD.^5^ Lp(a) concentration is primarily determined by the rates of production even though the catabolism of Lp(a) particles may play a role, as reflected by the kinetics of apo(a). The precise impact of genetic variation in apo(a) on the dynamics of Lp(a) particles in the circulation remains to be fully established, although there is a significant inverse association between the number of kringle‐IV (KIV) type 2 repeats in apo(a) and plasma Lp(a) concentrations. The number of KIV type 2 repeats determine at least 60% of the variation in plasma Lp(a) concentrations.^7^, ^8^ However, the association between apo(a) isoform size and Lp(a) kinetics in plasma is not well defined, partly because only a limited number of studies have been reported to date.^9^, ^10^ Some kinetic studies in humans suggest that variation in Lp(a) plasma concentration is primarily determined by the rates of production and not catabolism, and that Lp(a) production is inversely associated with apo(a) isoform size.^9^, ^10^, ^11^, ^12^, ^13^, ^14^ An association between apo(a) isoform size and the catabolism of Lp(a) particles has also been reported in some studies.^10^ Inconsistencies among the results of these kinetic studies may be partly because of small sample sizes.

While apoB is essential for the binding of LDL particles to the LDL receptor, necessary for cellular uptake and degradation by the liver, the role of the LDL receptor in Lp(a) particle catabolism remains unclear.^15^, ^16^, ^17^, ^18^ Experimental studies have demonstrated that the LDL receptor is involved in Lp(a) catabolism,^15^, ^16^, ^17^ but the findings have not been conclusive. Early radiolabeled kinetic studies showed no difference in the fractional catabolic rate (FCR) of Lp(a) between control subjects and patients with homozygous familial hypercholesterolemia, who lack hepatic LDL receptors, suggesting that the LDL receptor is not required in the catabolism of Lp(a).^18^ Statins and proprotein convertase subtilisin‐kexin type 9 inhibition enhance hepatic LDL receptor activity, but their effects on plasma Lp(a) concentration appear to differ.^19^ While statins alone show a modest or no effect on plasma Lp(a) concentration,^6^, ^20^, ^21^ proprotein convertase subtilisin‐kexin type 9 monoclonal antibody monotherapy, such as evolocumab, appears to lower plasma Lp(a) concentration by decreasing the production of Lp(a) particles,^6^, ^22^ particularly in subjects with elevated Lp(a).^23^ Whether variations in the apo(a) gene bear on the kinetics of Lp(a) particles and particularly against background maximal LDL‐cholesterol‐lowering therapy remains unknown. Knowledge of the determinants of the kinetics of Lp(a) and the response to therapies is fundamentally important to broaden our understanding of this complex, heritable cardiovascular risk factor and to provide a rationale for best use of new treatments.

To gain a better understanding of the association between apo(a) isoform size and Lp(a) kinetics in humans, we performed a kinetic study of Lp(a) particles using stable isotope labeling in a larger study of 63 apparently healthy individuals. The primary objective of the present study was to investigate the extent to which the plasma concentration of Lp(a) is determined by the production and catabolism of Lp(a) particles, as reflected by the kinetics of apo(a), under normal physiological conditions. The secondary objective was to examine the dependence of these associations on apo(a) isoform size and background therapy with atorvastatin and/or evolocumab therapy. To investigate the role of LDL receptor and related receptors in the catabolism of Lp(a) particles, we also examined associations between the catabolism of Lp(a) and the catabolism of other apoB‐100 containing lipoproteins, principally LDL particles also under normal physiological conditions.

## Materials and Methods

### Subjects

The present investigation was part of a larger tracer study examining the kinetic effects of atorvastatin and evolocumab on lipoprotein metabolism in healthy normolipidemic men (http://www.clinicaltrials.gov; NCT02189837).^6^, ^24^ The data that support the findings of this study are available from Amgen Inc (www.amgen.com) upon reasonable request. Pre‐intervention (baseline) data were used to examine the association between the plasma concentration, kinetics, and isoform size of apo(a) in available subjects. Postintervention data from the same cohort were also used to verify the association observed in the baseline data and to investigate these associations of apo(a) metabolism against background treatment with atorvastatin and/or evolocumab. Full details of the recruitment of subjects were published previously.^6^, ^24^ Briefly, we studied 63 healthy, normolipidemic white men aged 18 to 65 years with body mass index of 18 to 32 kg/m^2^, fasting plasma LDL‐cholesterol of ≥2.5 and <4.9 mmol/L and triglycerides of <1.7 mmol/L, (ie, within a population reference range that excluded those with significant dyslipidemia). None of the subjects had familial hypercholesterolemia or secondary hyperlipidemias (diabetes mellitus, chronic kidney disease, hypothyroidism, or were taking medications); further exclusion criteria were published previously.^6^, ^24^ All subjects were consuming isocaloric diets and did light‐to‐moderate exercise. The study was approved by a national ethics committee (Bellberry Ltd, Eastwood, South Australia); all subjects provided informed consent.

### Clinical Protocol

Eligible subjects were admitted to the site metabolic ward after a 14‐hour fast. They were studied in a semirecumbent position and allowed to drink only water. Venous blood was collected for laboratory measurements, and plasma volume was determined by multiplying body weight by 0.045. A single bolus of D3‐leucine (5 mg/kg of body weight) was administered intravenously within a 2‐minute period into an antecubital vein. Blood samples were taken at baseline and at 5, 10, 20, 30, and 40 minutes and at 1, 1.5, 2, 2.5, 3, 4, 5, 6, 8, and 10 hours after injection of the isotope. Subjects were then given a snack and discharged home. Additional fasting blood samples were collected in the morning on the following 4 days of the same week (ie, at 24, 48, 72, and 96 hours after injection of the isotope). All the procedures were repeated after the 8‐week intervention period (ie, placebo, atorvastatin, evolocumab, and atorvastatin plus evolocumab interventions) as previously described.^6^

### Isolation of Lipoproteins and Measurement of Isotopic Enrichment

Full details of methods, including quantification of very‐low density lipoprotein (VLDL)‐B‐100, intermediate‐density lipoprotein (IDL)‐apoB‐100, and LDL‐apoB‐100 and isolation and measurement of isotopic enrichment of apo(a), VLDL‐apoB‐100, IDL‐apoB‐100, and LDL‐apoB‐100 have been published elsewhere.^6^, ^24^ To avoid interference of Lp(a)‐apoB‐100 with the measurement of LDL‐apoB‐100, we removed Lp(a) from plasma before ultracentrifugation using an immunomagnetic isolation method (Dynabeads Protein G; Life Technologies, Victoria, Australia) with beads coated with a goat polyclonal antibody (immunoglobulin G) to human Lp(a) (Advy Chemical Ltd, Mumbai, India).^24^

### Quantification of Lp(a) and apo(a)

Plasma apo(a) concentration was determined following a standardized sample trypsin digestion procedure and liquid chromatography–mass spectrometry with a specific target peptide, LFLEPTQADIALL.^22^ Full details have been published elsewhere.^6^ This liquid chromatography–mass spectrometry method was validated against a reference immunoassay based on a monoclonal antibody directed to apo(a) (Northwest Lipid Metabolism and Diabetes Research Laboratories, University of Washington, Seattle, WA). The value to the assay calibrator was assigned by amino acid analysis using a purified Lp(a) and the values in nmol/L refer to the moles of apo(a).^25^ The values obtained by these 2 methods were closely correlated at baseline and after intervention (*r*=0.945 and 0.967, respectively, *P*<0.001 for both), because they both measure apo(a) without the impact of apo(a) size polymorphism. Variability in the difference between the 2 assays is because of the differences in the analytical principles used and/or use of different assay calibrators.

### Determination of Apo(a) Isoform Size

Apo(a) isoform size was determined by a high‐sensitive SDS‐agarose gel electrophoresis followed by immunoblotting (Northwest Lipid Metabolism and Diabetes Research Laboratories, University of Washington, Seattle, WA) and the apo(a) isoforms are expressed in terms of the respective number of KIV repeats as previously reported.^26^ The majority of individuals have 2 circulating apo(a) of different sizes and therefore, the predominantly expressed apo(a) isoform was used for statistical analysis.

### Compartmental Models and Calculation of Kinetic Parameters

Compartmental analysis, using the SAAM II program (The Epsilon Group, VA), was used to fit a model to the isotopic enrichment data for apo(a), VLDL‐apoB‐100, IDL‐apoB‐100, and LDL‐apoB‐100.^6^, ^24^ The apo(a) enrichment data were modeled using a single‐pool model as previously described.^6^ Briefly, plasma leucine kinetics were described by a 4‐compartment model, which was connected to intrahepatic delay compartments (compartments 5 and 6) that accounted for the synthesis and secretion of Lp(a)‐apo(a) and Lp(a)‐apoB‐100, respectively, with compartments 7 and 8 describing the plasma kinetics of Lp(a)‐apo(a) and Lp(a)‐apoB‐100. The FCR of Lp(a)‐apo(a) was estimated after fitting the model to the apo(a) tracer/tracee ratio data. The hepatic production rate (PR) of Lp(a)‐apo(a) was calculated as the product of FCR and pool size of Lp(a)‐apo(a). Pool size was derived by multiplying plasma volume and the plasma Lp(a)‐apo(a) concentration measured by liquid chromatography–mass spectrometry.

### Statistical Analyses

All analyses were performed using SPSS 21 (SPSS, Inc, Chicago). Group characteristics were compared by *t* tests, after logarithmic transformation of skewed variables where appropriate. The subjects were also grouped according to apo(a) isoform sizes with reference to a cut‐off of KIV repeats ≤22; subjects with ≤22 KIV repeats (ie, smaller apo(a) isoform sizes) have been shown to be associated with an increased risk for ASCVD.^27^, ^28^ Associations were examined by Pearson's correlational analyses using all baseline data before treatments (n=63) and data only on active treatments (ie, atorvastatin, evolocumab, atorvastatin plus evolocumab, n=47).^6^, ^24^ The decision to undertake the second, more restricted analyses was to explore whether the baseline associations were similar to those active treatments, where LDL‐cholesterol was lowered. Stepwise linear regression was used to analyze whether apo(a) PR or apo(a) FCR was the best predictor of plasma apo(a) concentration. For multiple regression models using all baseline data before treatments and data on active treatments, we selected variables (ie, apo(a) PR, apo(a) FCR, apo(a) isoform size, LDL‐apoB‐100 FCR) that could be causally related with the plasma concentration of apo(a). We did not measure LDL receptor activity directly, but inferred it from the FCR of LDL‐apoB‐100 in the regression models to investigate the impact of LDL receptor activity on Lp(a) kinetics. The type of active treatment (ie, atorvastatin, evolocumab, atorvastatin plus evolocumab) was also included as a predictor in the multivariable regression models to assess their effects on the predictors of apo(a) concentration. All independent variables were entered into the regression models at the same time. Statistical significance was defined at the 5% level using a 2‐tailed test.

## Results

### Baseline (Pretreatment) Analyses

The anthropometric, biochemical, and lipoprotein kinetic characteristics of the 63 white male subjects at baseline (ie, pretreatment) are summarized in Table 1. They were on average 33 years old, nonobese, normotensive, nondiabetic, and had normal plasma lipid and lipoprotein profiles. Thirty‐six subjects had a small apo(a) isoform at a cut‐off of KIV repeats ≤22.

**Table 1:** Anthropometric and Biochemical Characteristics and Kinetic Parameters of Lp(a)‐apo(a) and LDL‐apoB‐100 at Baseline in the 63 Subjects Studied

Characteristics	Mean±SD	Range
Age, y	33±10	18–57
Systolic blood pressure, mm Hg	124±10	97–147
Diastolic blood pressure, mm Hg	77±10	45–99
Body mass index, kg/m^2^	25±3	19–30
Glucose, mmol/L	5.3±0.4	4.4–6.5
Insulin, U/L	6.0±2.9	2.7–17
HOMA score	1.4±0.7	0.63–4.46
Cholesterol, mmol/L	4.7±0.6	3.1–6.2
Triglycerides, mmol/L^a^	0.87	0.80–0.94
HDL‐cholesterol, mmol/L	1.2±0.3	0.75–1.9
LDL‐cholesterol, mmol/L	3.0±0.46	2.0–4.3
ApoA‐I, g/L	1.4±0.21	1.0–1.9
ApoB, g/L	0.85±0.12	0.55–1.1
Predominant apo(a) isoform KIV repeats^a^	22	15–34
Apo(a), nmol/L^a^	22	18–27
Apo(a) FCR, pool/day^a^	0.40	0.36–0.45
Apo(a) PR, nmol/kg per day^a^	0.39	0.32–0.49
LDL‐apoB‐100, mg/L	458±143	181–863
LDL‐apoB‐100 FCR, pool/day	0.46±0.13	0.26–0.83
LDL‐apoB‐100 PR, mg/kg per day	9.4±3.9	3.1–21.3

Apo indicates apolipoprotein; FCR, fractional catabolic rate; HDL, high‐density lipoprotein; HOMA, homeostasis model assessment; KIV, kringle‐IV; LDL, low‐density lipoprotein; PR, production rate.

a Values expressed as geometric mean (95% CI).

#### Univariate regression analyses

**Figure 1:**
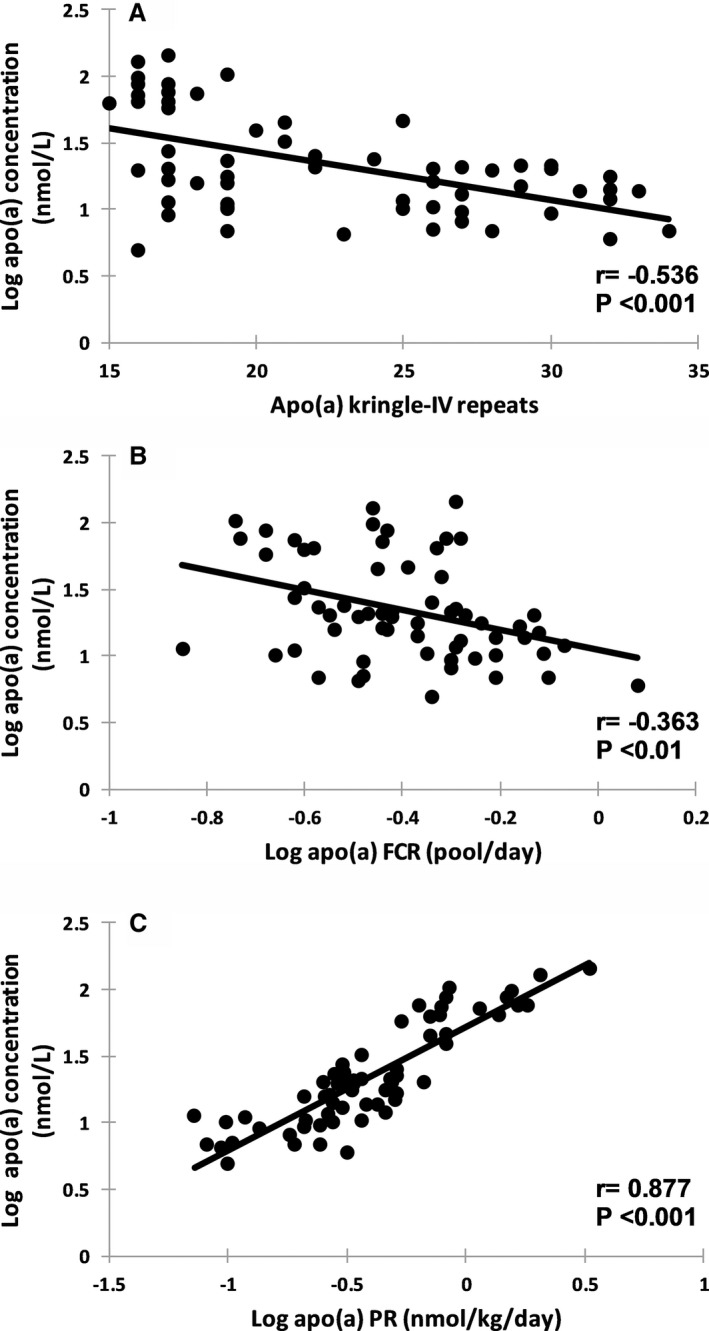
Association between plasma apolipoprotein(a) (apo(a)) concentration and apo(a) isoform size (**A**), apo(a) fractional catabolic rate (FCR) (**B**), and apo(a)‐production rate (PR) (**C**) at baseline in the 63 subjects.

Table 2 shows the associations of plasma concentration, FCR, and PR of apo(a) with lipids, lipoproteins concentrations, and the kinetic parameters of LDL‐apoB‐100 at baseline in the 63 subjects. In univariate analysis, plasma apo(a) concentration was significantly and inversely correlated with apo(a) isoform size (*r*=−0.536, *P*<0.001) and apo(a) FCR (*r*=−0.363, *P*<0.01), and positively with apo(a) PR (*r*=0.877, *P*<0.001) (Figure 1A through 1C). Apo(a) isoform size was significantly and positively associated with apo(a) FCR (*r*=0.618, *P*<0.001) and inversely with apo(a) PR (*r*=−0.251, *P*<0.05). In stepwise regression including apo(a) PR and apo(a) FCR, apo(a) PR was the best predictor in determining apo(a) concentration (adjusted *R* ^2^=0.70, *P*<0.001) in the 63 subjects. As seen in Table 2, plasma apo(a) concentration was not significantly associated with the FCRs or PRs of LDL‐apoB‐100 (*P*>0.05 for all). There was also no significant association between the FCR of apo(a) and LDL‐apoB‐100 (*r*=0.195, *P*>0.05). Apo(a) PR was not significantly associated with the PR of LDL‐apoB‐100 (*r*=−0.134, *P*>0.05). There was no significant association between apo(a) FCR and the FCRs of VLDL‐apoB‐100 (*r*=0.188, *P*>0.05) and IDL‐apoB‐100 (*r*=0.147, *P*>0.05).

**Table 2:** Associations (Pearson Correlation Coefficients) of Plasma Concentration, FCR, and PR of apo(a) With Plasma Lipids, Lipoproteins, and LDL‐apoB‐100 Kinetics at Baseline in the Subjects Studied

Characteristics	Lp(a)‐apo(a)		
	Concentration	FCR	PR
Cholesterol, mmol/L	−0.022	0.092	−0.022
Triglycerides, mmol/L	−0.065	0.031	−0.053
HDL‐cholesterol, mmol/L	−0.078	0.111	−0.026
LDL‐cholesterol, mmol/L	0.041	−0.088	−0.001
ApoA‐I, g/L	−0.087	0.024	−0.081
ApoB, g/L	0.033	−0.066	0.001
Predominant apo(a) isoform KIV	−0.536^a^	0.618^a^	−0.251^b^
Apo(a), nmol/L		−0.363^c^	0.877^a^
Lp(a)‐apo(a) FCR, pool/day	−0.363^c^		0.130
Lp(a)‐apo(a) PR, nmol/kg per day	0.877^a^	0.130	
LDL‐apoB‐100, mg/L	−0.225	0.100	−0.188
LDL‐apoB‐100 FCR, pool/day	−0.139	0.195	−0.047
LDL‐apoB PR‐100, mg/kg per day	−0.225	0.203	−0.134

Apo indicates apolipoprotein; FCR, fractional catabolic rate; HDL, high‐density lipoprotein; KIV, kringle‐IV; LDL, low‐density lipoprotein; PR, production rate.

a *P*<0.001.

b *P*<0.05.

c *P*<0.01.

#### Multivariable regression analyses

Table 3 gives multiple regression showing that apo(a) PR (β‐coefficient 0.792, *P*<0.001) and apo(a) isoform size (β‐coefficient −0.331, *P*<0.001) were significant predictors of the plasma concentration of apo(a) after adjusting for LDL‐apoB‐100 FCR (adjusted *R* ^2^=0.869, *P*<0.001). In multiple regression analysis including apo(a) FCR, apo(a) isoform size and LDL‐apoB‐100 FCR (Table 4), apo(a) isoform size (β‐coefficient −0.501, *P*=0.01) was the only significant predictor of the plasma concentration of apo(a) (adjusted *R* ^2^=0.253, *P*<0.001). The data shown in Tables 3 and 4 were also similar after including age of the subject as a predictor in the regression models (data not shown).

**Table 3:** Multiple Linear Regression Analyses in the Baseline Data Showing apo(a) PR, apo(a) Isoform Size, and LDL‐apoB‐100 FCR as Predictors of apo(a) Concentration

Predictor Variable	Partial *R* ^2^	Standardized β‐Coefficient	Standard Error	*P* Value
Apo(a) PR	0.588	0.792	0.051	<0.001
Apo(a) isoform size	0.097	−0.331	0.003	<0.001
LDL‐apoB‐100 FCR	0.001	−0.025	0.003	0.601
Adjusted *R* ^2^=0.869	*P*<0.001			

Apo indicates apolipoprotein; FCR, fractional catabolic rate; LDL, low‐density‐lipoprotein; PR, production rate.

**Table 4:** Multiple Linear Regression Analyses in the Baseline Data Showing apo(a) FCR, apo(a) Isoform Size, and LDL‐apoB‐100 FCR as Predictors of apo(a) Concentration

Predictor Variable	Partial *R* ^2^	Standardized β‐Coefficient	Standard Error	*P* Value
Apo(a) FCR	0.016	−0.051	0.288	0.717
Apo(a) isoform size	0.152	−0.501	0.01	0.001
LDL‐apoB‐100 FCR	0.001	−0.013	0.332	0.91
Adjusted *R* ^2^=0.253	*P*<0.001			

Apo indicates apolipoprotein; FCR, fractional catabolic rate; LDL, low‐density lipoprotein.

#### Analyses according to apo(a) isoform size

**Figure 2:**
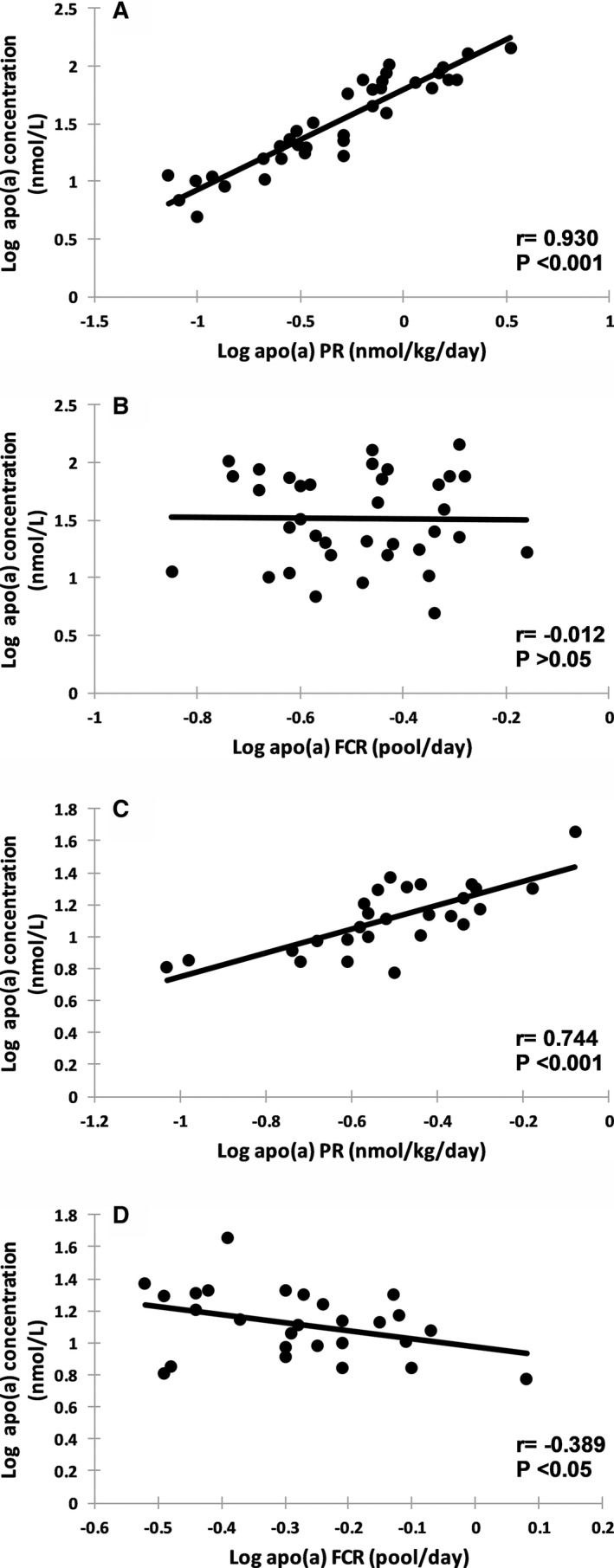
Association between plasma apolipoprotein(a) (apo(a)) concentration and apo(a) production rate (PR) and apo(a) fractional catabolic rate (FCR) at baseline in subjects with smaller apo(a) isoforms ≤22 kringle‐IV (KIV) (n=36, **A** and **B**, respectively) and larger apo(a) isoforms >22 KIV (n=27, **C** and **D**, respectively).

Table 5 shows the plasma apo(a) concentrations and corresponding apo(a) kinetic parameters in the subjects grouped according to apo(a) isoform size. Compared with subjects with larger apo(a) isoform sizes (ie, KIV repeats >22, n=27), there was significant increase in plasma apo(a) concentration (*P*<0.001) in subjects with small apo(a) isoforms (ie, KIV repeats ≤22, n=36). Additionally, subjects with smaller apo(a) isoforms had significantly higher apo(a) PR (*P*<0.05) and reduced apo(a) FCR (*P*<0.01) compared with those with larger apo(a) isoform sizes. The concentration, FCR, and PR of LDL‐apoB‐100 were not significantly different between the 2 groups (*P*>0.05 for all). Similar differences were observed when the subjects were grouped according to apo(a) concentration at a geometric mean cut‐off of 22 nmol/L (Table 6). Because we only studied subjects randomly selected from the community, choosing higher cut‐off values for apo(a) to classify groups would result in too few subjects in the “elevated” group to make the statistical analysis valid (eg, 70 nmol/L, n=11; 100 nmol/L, n=3). There were also no significant differences in the concentration, FCR, and PR of VLDL‐apoB‐100 and IDL‐apoB‐100, nor plasma lipid and lipoprotein concentrations, between the larger and smaller isoform size groups (Table 7). In subjects with smaller apo(a) isoform sizes, plasma apo(a) concentration was significantly and positively correlated with apo(a) PR (*r*=0.930, *P*<0.001), but not with apo(a) FCR (*r*=−0.012, *P*>0.05) (Figure 2A and 2B). The FCR and PR of apo(a) was significantly and positively correlated (*r*=0.359, *P*<0.05). In subjects with larger apo(a) isoform sizes, plasma apo(a) concentration was significantly and positively correlated with apo(a) PR (*r*=0.744, *P*<0.001), and inversely with apo(a) FCR (*r*=−0.389, *P*<0.05) (Figure 2C and 2D). The FCR and PR of apo(a) was positively correlated (*r*=0.356), but this association did not reach statistical significance (*P*=0.07) in these subjects. There were no significant associations of the concentration, FCR, and PR of apo(a) with the kinetics of LDL‐apoB‐100 in subjects with smaller or larger apo(a) isoform sizes (data not shown).

**Table 5:** Plasma apo(a) and the Kinetics of apo(a) and LDL‐apoB‐100 at Baseline in the 63 Subjects Grouped According to apo(a) Isoform Size

Characteristics	Apo(a) Isoform Size	
	Small (KIV Repeats ≤22)	Large (KIV Repeats >22)
Predominant apo(a) isoform KIV	18 (17–19)^a^	28 (27–29)
Apo(a), nmol/L	33 (24–44)^a^	13 (11–16)
Apo(a) FCR, pool/day	0.32 (0.29–0.37)^b^	0.53 (0.46–0.61)
Apo(a) PR, nmol/kg per day	0.47 (0.34–0.66)^c^	0.31 (0.26–0.38)
LDL‐apoB‐100, mg/L	455±150	461±137
LDL‐apoB‐100 FCR, pools/day	0.43±0.13	0.49±0.13
LDL‐apoB‐100 PR, mg/kg per day	8.9±4.3	9.9±3.2

Values expressed as mean±SD or geometric mean (95% CI). Apo indicates apolipoprotein; FCR, fractional catabolic rate; KIV, kringle‐IV; LDL, low‐density lipoprotein; PR, production rate.

a *P*<0.001.

b *P*<0.01.

c *P*<0.05.

**Table 6:** Plasma apo(a) and the kinetics of apo(a) and LDL‐apoB‐100 at baseline in the 63 subjects grouped according to apo(a) level

Characteristics	Apo(a) levels	
	Low (≤ 22 nmol/L)	High (>22 nmol/L)
Predominant apo(a) isoform KIV	24 (22–26)^a^	18 (17–19)
Apo(a), nmol/L	12 (11–14)‡	56 (45–72)
Apo(a) FCR, pool/day	0.45 (0.40–0.52)^b^	0.33 (0.28–0.38)
Apo(a) PR, nmol/kg/day	0.25 (0.21–30)^a^	0.83 (0.63–1.08)
LDL‐apoB‐100, mg/L	481±146	420±132
LDL‐apoB‐100 FCR, pools/day	0.46±0.13	0.45±0.13
LDL‐apoB‐100 PR, mg/kg/day	9.8±3.7	8.6±4.1

Values expressed as mean±SD or geometric mean (95% confidence interval); Apo, apolipoprotein; FCR, fractional catabolic rate; LDL, low‐density lipoprotein; PR, production rate.

a *P* < 0.01.

b *P* < 0.001.

**Table 7:** Plasma Lipids, Lipoproteins, and the Kinetics of VLDL‐apoB Lp(a)‐apo(a) and LDL‐apoB‐100 at Baseline in the 63 Subjects Grouped According to apo(a) Isoform Size

Characteristics	Apo(a) Isoform Size	
	Small (KIV Repeats ≤22)	Large (KIV Repeats >22)
Cholesterol, mmol/L	4.7±0.57	4.7±0.70
Triglycerides, mmol/L	0.90±0.28	0.93±0.37
HDL‐cholesterol, mmol/L	1.2±0.26	1.3±0.31
LDL‐cholesterol, mmol/L	3.1±0.47	3.1±0.44
ApoA‐I, g/L	1.2±0.26	1.3±0.31
ApoB, g/L	0.85±0.12	0.85±0.12
VLDL‐apoB‐100, mg/L	52±24	49±26
VLDL‐apoB‐100 FCR, pools/day	10±4.9	11±5.1
VLDL‐apoB‐100 PR, mg/kg per day	21±8.1	21±8.5
IDL‐apoB‐100, mg/L	36±11	38±16
IDL‐apoB‐100 FCR, pools/day	7.0±3.7	7.2±3.0
IDL‐apoB‐100 PR, mg/kg per day	11±5.1	11±3.6

There were no statistically significant differences between the 2 groups on any of the variables shown in the table. Apo indicates apolipoprotein; FCR, fractional catabolic rate; HDL, high‐density lipoprotein; IDL, intermediate‐density lipoprotein; KIV, kringle‐IV; LDL, low‐density lipoprotein; PR, production rate; VLDL, very‐low‐density lipoprotein.

Values expressed as mean±SD.

### Analyses on Active Treatments (Atorvastatin, Evolocumab, Atorvastatin Plus Evolocumab)

The full set of results of the effects of atorvastatin and evolocumab, alone and in combination on plasma lipid and lipoprotein concentrations, and on the kinetics of LDL‐apoB‐100 and Lp(a)‐apo(a) kinetics have been previously reported.^6^ Briefly, both atorvastatin and evolocumab, monotherapy or in combination, decreased plasma concentrations of total cholesterol, LDL‐cholesterol, and apoB‐100, as well as the concentration of LDL‐apoB‐100 by accelerating their catabolism. Evolocumab monotherapy, but not atorvastatin, lowered the plasma Lp(a) pool size by decreasing the production of Lp(a) particles. In combination with atorvastatin, evolocumab lowered the plasma Lp(a) pool size by accelerating the catabolism of Lp(a) particles.

#### Univariate regression analyses

**Figure 3:**
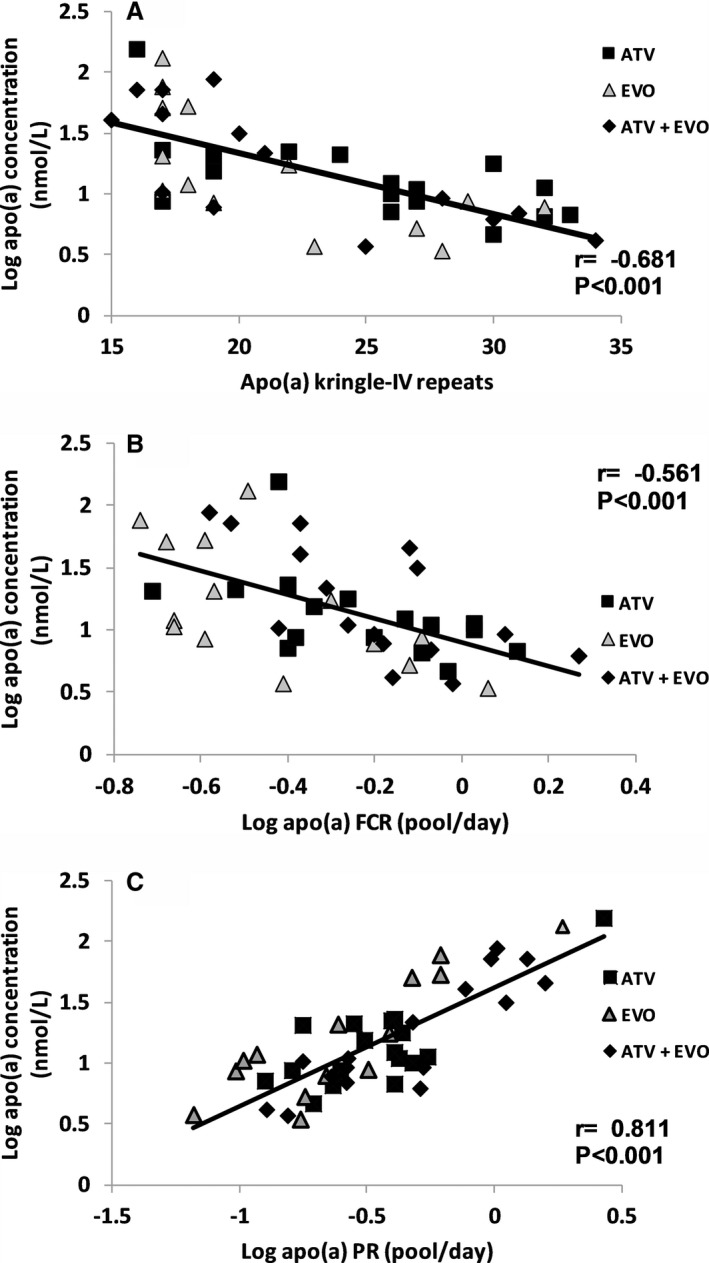
Association between plasma apolipoprotein(a) (apo(a)) concentration and apo(a) isoform size (**A**), apo(a) fractional catabolic rate (FCR) (**B**), and apo(a) production rate (PR) (**C**) in the 47 subjects on active treatments. ATV indicates atorvastatin; EVO, evolocumab.

In a pooled analysis using post‐treatment data (n=47), plasma apo(a) concentration was significantly and inversely associated with apo(a) isoform size (*r*=−0.681, *P*<0.001; Figure 3A) and the FCR of apo(a) (*r*=−0.561, *P*<0.001; Figure 3B), and positively with the PR of apo(a) (*r*=0.811, *P*<0.001; Figure 3C). These on‐treatment observations confirmed our baseline findings on apo(a) metabolism (Table 2 and Figure 1).

#### Multivariable regression analyses

In multiple regression analyses (Table 8), apo(a) PR and apo(a) isoform size were significant predictors of the plasma apo(a) concentration after adjusting for LDL‐apoB‐100 FCR, and type of treatment (ie, atorvastatin, evolocumab, atorvastatin plus evolocumab) (β‐coefficient 0.678 and −0.456, respectively, *P*<0.001 for both). Apo(a) isoform size, but not apo(a)‐FCR, was a significant predictor of the plasma apo(a) concentration after adjusting for LDL‐apoB‐100 FCR, type of treatment (Table 9, β‐coefficient −0.576, *P*<0.01). The results in Tables 8 and 9 held after adjusting for age of the subject as an additional predictor (data not shown).

**Table 8:** Multiple Linear Regression Analyses Using Data Pooled From Subjects Receiving Active Treatments (n=47) Showing apo(a) PR and apo(a) Isoform Sizes as Predictors of apo(a) Concentrations

Predictor Variable	Partial *R* ^2^	Standardized β‐Coefficient	Standard Error	*P* Value
Apo(a) PR	0.413	0.678	0.068	<0.001
Apo(a) isoform size	0.143	−0.456	0.005	<0.001
LDL‐apoB‐100 FCR	0.004	−0.09	0.046	0.234
Type of treatment^a^	0.001	−0.033	0.036	0.651
Adjusted *R* ^2^=0.869	*P*<0.001			

Models including apo(a) PR, apo(a) isoform sizes, LDL‐apoB‐100 FCR, and type of treatment as predictor variables. Apo indicates apolipoprotein; FCR, fractional catabolic rate; LDL, low‐density lipoprotein; PR, production rate.

a Type of treatment includes atorvastatin, evolocumab and atorvastatin plus evolocumab.

**Table 9:** Multiple Linear Regression Analyses Using Data Pooled From Subjects Receiving Active Treatments (n=47) Showing apo(a) Isoform Size as a Predictor of apo(a) Concentrations

Predictor Variable	Partial *R* ^2^	Standardized β‐Coefficient	Standard Error	*P* Value
Apo(a) FCR	0.001	−0.115	0.301	0.516
Apo(a) isoform size	0.131	−0.576	0.013	0.003
LDL‐apoB‐100 FCR	0.001	−0.049	0.098	0.761
Type of treatment^a^	0.001	0.029	0.076	0.847
Adjusted *R* ^2^=0.422	*P*<0.001			

Models including apo(a) FCR, apo(a) isoform size, LDL‐apoB‐100 FCR, and type of treatment as predictor variables. Apo indicates apolipoprotein; FCR, fractional catabolic rate; LDL, low‐density lipoprotein.

a Type of treatment includes atorvastatin, evolocumab and atorvastatin plus evolocumab.

As seen in Table 10, apo(a) PR and apo(a) isoform size remained significant predictors of plasma apo(a) concentration after adjusting for LDL‐apoB‐100 FCR in the atorvastatin (β‐coefficient 0.651 and −0.545, respectively, *P*<0.001 for both), evolocumab (β‐coefficient 0.730 and −0.431, respectively, *P*<0.001 for both), and atorvastatin plus evolocumab treatment groups (β‐coefficient 0.683 and −0.271, respectively, *P*<0.05 for both). Apo(a) isoform size was a negative predictor of plasma apo(a) concentration (Table 11) after adjusting for LDL‐apoB‐100 FCR in the atorvastatin (β‐coefficient −0.632, *P*<0.05), evolocumab (β‐coefficient −0.705, *P*=0.238), and atorvastatin plus evolocumab treatment groups (β‐coefficient −0.587, *P*<0.05); however, the *P* value did not reach statistical significance in the evolocumab alone group. The lack of statistical significance in this analysis reflects the small sample size of this treatment group. Accordingly, in post‐treatment data pooled from the atorvastatin (n=17) and evolocumab alone (n=14) groups, apo(a) isoform size was a statistically significant predictor of plasma apo(a) concentration (β‐coefficient −0.587, *P*<0.05).

**Table 10:** Multiple Linear Regression Analyses Using Data Pooled From Subjects Receiving Active Treatments (n=47) Showing apo(a) PR and apo(a) Isoform Sizes as Predictors of apo(a) Concentrations

Predictor Variable	Partial *R* ^2^	Standardized β‐Coefficient	Standard Error	*P* Value
Atorvastatin group (n=17)				
Apo(a) PR	0.376	0.651	0.133	<0.001
Apo(a) isoform size	0.277	−0.545	0.007	<0.001
LDL‐apoB‐100 FCR	0.004	0.065	0.204	0.574
Adjusted *R* ^2^=0.817; *P*<0.001				
Evolocumab group (n=14)				
Apo(a) PR	0.493	0.731	0.086	<0.001
Apo(a) isoform size	0.111	−0.431	0.008	<0.001
LDL‐apoB‐100 FCR	0.005	−0.088	0.134	0.322
Adjusted *R* ^2^=0.944; *P*<0.001				
Atorvastatin plus evolocumab group (n=16)				
Apo(a) PR	0.301	0.683	0.144	<0.001
Apo(a) isoform size	0.038	−0.271	0.491	0.049
LDL‐apoB‐100 FCR	0.031	−0.202	0.426	0.072
Adjusted *R* ^2^=0.881; *P*<0.001				

Models including apo(a) PR, apo(a) isoform size and LDL‐apoB‐100 FCR as predictor variables. Apo indicates apolipoprotein; FCR, fractional catabolic rate; LDL, low‐density lipoprotein; PR, production rate.

**Table 11:** Multiple Linear Regression Analyses Using Data Pooled From Subjects Receiving Active Treatments (n=47) Showing apo(a) Isoform Size as a Predictor of apo(a) Concentrations

Predictor Variable	Partial *R* ^2^	Standardized β‐Coefficient	Standard Error	*P* Value
Atorvastatin group (n=17)				
Apo(a) FCR	0.005	−0.101	0.434	0.729
Apo(a) isoform size	0.178	−0.632	0.957	0.048
LDL‐apoB‐100 FCR	0.078	0.283	0.355	0.175
Adjusted *R* ^2^=0.401; *P*<0.05				
Evolocumab group (n=14)				
Apo(a) FCR	0.003	0.138	1.102	0.814
Apo(a) isoform size	0.081	−0.705	2.723	0.238
LDL‐apoB‐100 FCR	0.017	−0.174	0.477	0.576
Adjusted *R* ^2^=0.341; *P*=0.069				
Atorvastatin plus evolocumab group (n=16)				
Apo(a) FCR	0.032	−0.275	0.551	0.322
Apo(a) isoform size	0.195	−0.587	0.915	0.026
LDL‐apoB‐100 FCR	0.001	−0.006	0.959	0.981
Adjusted *R* ^2^=0.546; *P*<0.01				

Models including apo(a) FCR, apo(a) isoform size, and LDL‐apoB‐100 FCR as predictor variables. Apo indicates apolipoprotein; FCR, fractional catabolic rate; LDL, low‐density lipoprotein.

## Discussion

Our major finding was that in healthy normolipidemic white men, plasma apo(a) concentration, which reflects Lp(a) particle number, was chiefly determined by the rate of production of lipoprotein particles independent of apo(a) isoform size and treatment with atorvastatin and evolocumab. Subjects with smaller apo(a) isoform sizes had significantly elevated apo(a) concentration because of elevated production and reduced catabolism of apo(a). Apo(a) PR was, however, more strongly associated with apo(a) concentration than the apo(a) fractional catabolic rate. We also found no significant association under normal physiological conditions between the FCRs of apo(a) and LDL‐apoB‐100 in normolipidemic white men.

### Previous Studies of Lp(a) Particle Kinetics

Early radioisotopic studies in normal individuals and familial hypercholesterolemia patients have suggested that Lp(a) concentration is largely determined by the PR of Lp(a) with no significant association with FCR.^7^, ^29^ Rader et al demonstrated that the PR of Lp(a) was greater in subjects with smaller isoform sizes than those with larger isoform sizes, but there was no difference in Lp(a) catabolism.^9^ Recent kinetic studies using endogenous stable isotope labeling have not shown consistent associations between Lp(a) concentration, apo(a) isoform sizes, and Lp(a) kinetics.^11^, ^12^, ^13^, ^14^ This is probably because of differences in subject characteristics, sample size, and study designs. In a postprandial study of normal individuals, Jenner et al found that plasma Lp(a) concentration was positively associated with apo(a) PR and inversely with apo(a) FCR.^10^ We have extended previous studies by using a larger sample size to examine the kinetics of apo(a) in relation to apo(a) isoform size and background LDL‐cholesterol‐lowering therapies under normal fasting physiological condition. We also examined the association between the kinetics of apo(a) and apoB‐100 in VLDL, IDL, and LDL particles.

### Apo(a) Isoform Size and Apo(a) Production

It is generally considered that plasma apo(a) concentration is mainly determined by the production of apo(a), which is in turn determined by apo(a) isoform size.^7^, ^9^ Accordingly, we found that plasma apo(a) concentration was significantly and positively associated with apo(a) PR, and inversely with apo(a) isoform size, irrespective of LDL‐apoB‐100 FCR and background LDL‐cholesterol‐lowering therapies. Our findings are supported by several lines of experimental evidence. First, the size of the apo(a) transcripts is inversely associated with hepatic apo(a) mRNA concentration,^30^ and by implication apo(a) production. Second, larger apo(a) isoforms have been shown to have a longer retention time in the endoplasmic reticulum and probably greater intracellular apo(a) proteasome degradation, resulting in a less efficient secretion from hepatocytes.^31^ We also demonstrated that the significant association between apo(a) concentration and apo(a) PR was observed for all apo(a) isoform sizes. This reinforces the role of apo(a) PR as the main determinant of plasma apo(a) concentration. In our multivariable regression model (Table 3), we found that both apo(a) PR and apo(a) isoform size were independent predictors of plasma apo(a) concentration. This suggests that other factors beyond apo(a) size may regulate apo(a) production. For example, experimental studies have demonstrated that the availability of apoB for coupling to apo(a) could determine the formation of an Lp(a) particle.^32^ However, we found no significant association between LDL‐apoB‐100 levels and apo(a) PR at the baseline data (data not shown). Hence, the hypothesis that apoB availability is rate limiting for the formation of Lp(a) particle remains to be further investigated in humans. As discussed later, that apo(a) isoform size independently predicted the plasma concentration of apo(a) (Table 3) suggests a direct role of apo(a) isoform size in regulating the removal of Lp(a) particles.

### Apo(a) Isoform Size and Apo(a) Catabolism

The cellular and molecular mechanisms responsible for Lp(a) catabolism are not fully known. Previous studies in transgenic mice have demonstrated that apo(a) is an important ligand for Lp(a) uptake by the liver.^33^ There is also some evidence that apo(a) with small and large isoform sizes have different binding affinities to the LDL receptor and other receptors for Lp(a) catabolism.^34^ Hence, it is plausible that apo(a) isoform may influence the removal of Lp(a) from plasma. Consistent with it, we found that apo(a) isoform size was positively associated with apo(a) FCR. The diminished association between plasma apo(a) concentration and FCR after adjusting for apo(a) isoform (Table 4) may reflect a dual role of apo(a) isoform size in regulating the PR and FCR of apo(a).^7^, ^9^ In the present study, subjects with smaller apo(a) isoform sizes had lower apo(a) FCR compared with those with larger sizes. The precise reason is unclear. Previous experimental data suggest that Lp(a) can be preferably catabolized via non‐LDL receptor pathways involving other receptors, such as VLDL receptor, LDL receptor–related protein 1, megalin/gp330, scavenger receptor class B type 1 (SR‐B1), and plasminogen receptors.^5^, ^35^ Lp(a) particles with larger isoform size have also been shown to be more effectively removed by the LDL receptor independent routes.^34^ Collectively, our findings suggest that Lp(a) particles with smaller apo(a) isoform are less preferably removed via non‐LDL receptor pathways under normal physiological condition. The lack of significant association between the FCRs of apo(a) and LDL‐apoB‐100 in the baseline data is consistent with the notion that the LDL receptor may not play a major role for the catabolism of Lp(a) particles under physiological conditions. The role of the LDLR receptor in Lp(a) catabolism in patients with high LDL‐cholesterol and Lp(a) who are treated with statins and proprotein convertase subtilisin‐kexin type 9 inhibitors remains to be established.^5^, ^18^, ^35^ Despite the FCR of apo(a) being significantly lower in subjects with smaller apo(a) isoform sizes, we found that in this setting, the FCR of apo(a) was positively correlated with the PR of apo(a). This may reflect a balancing, feed‐forward mechanism in response to increased production of apo(a) in these subjects.^36^ The contribution of other genetic and physiological factors, such as APOE genotype and renal function, to the FCR and plasma concentrations of apo(a) merits further investigations.^37^, ^38^

### Apo(a) and LDL‐apoB Catabolism

The role of the LDL receptor in Lp(a) clearance remains controversial. Early radioisotopic studies showed that the LDL receptor is not required for normal catabolism of Lp(a).^18^ Equilibrium binding studies showed that the affinity and maximum binding capacity to LDL receptor are lower for Lp(a) than for LDL.^16^ Reduced binding of Lp(a) particles to LDL receptors may be caused by camouflaging by the apo(a) moiety of the ligand‐binding domain of apoB.^33^ In the present study, we found no significant association between the FCRs of apo(a) and LDL‐apoB‐100. This observation is supported by our recent finding that in healthy, normolipidemic individuals, atorvastatin increased LDL‐apoB‐100 FCR without significantly affecting the plasma concentration or FCR of apo(a).^6^, ^24^ In the same study,^6^ evolocumab, but not atorvastatin, monotherapy lowered the plasma Lp(a) pool size by decreasing the production of Lp(a) particles. We also found that evolocumab in combination with atorvastatin reduced apo(a) concentration by increasing the FCR of Lp(a)‐apo(a). The increase in the FCR of Lp(a)‐apo(a) in the combination therapy group was directly correlated with the increase in LDL‐apoB FCR. Hence, we cannot exclude the possibility that the LDL receptor pathway plays a major role when LDL receptor expression and activity are maximally upregulated.^6^ The effects of other cellular receptors, including VLDL receptor, LDL receptor–related protein 1, SR‐BI, and plasminogen receptor, on Lp(a) catabolism also merit further investigation.

### Strengths and Weaknesses

A strength of our study is that we used a well‐validated compartment model to describe the kinetics of apo(a) and LDL‐apoB‐100 in a large sample of normolipidemic men. The selection of healthy, insulin sensitive, normolipidemic subjects ensured that the LDL receptor (or other related receptor) pathway was fully functional and plasma Lp(a) transport physiological. Our on‐treatment observation confirmed and reinforced our baseline findings on apo(a) metabolism. Although strictly exploratory, the importance of the on‐treatment analyses relate to improved knowledge of the kinetic determinants of Lp(a) metabolism in the setting of future add‐on therapies that target the production of Lp(a) particles.^39^ However, our study does have limitations. The inferences based on correlational analyses in the individual on‐treatment groups are tenuous because of small sample size, but we overcome this by pooling individual data from all 3 active treatment groups. For calculating apo(a) pool sizes, we did not quantitate non‐Lp(a)‐associated apo(a) and inferred it from total plasma apo(a) concentration at baseline and after intervention. Given the very minimal contribution of non‐Lp(a)‐associated apo(a) (<5%) to the total plasma apo(a) concentration,^40^ we consider that the former would not impact significantly on our findings. Whether statin or evolocumab therapy alter non‐Lp(a)‐associated apo(a) concentration merits further investigation.

We did not measure the kinetics of both apo(a) isoforms in all our subjects partly because the gel separation method we use does not provide enough concentration of the minor apo(a) isoform to be able to precisely measure the corresponding isotopic enrichment; this especially applies to the majority of the subjects who had low total plasma apo(a) concentrations. The estimation of Lp(a) kinetics based on the predominant isoform may be particularly confounded in subjects with widely different isoform sizes (eg, >8 KIV repeats). However, we consider that our kinetic data would reasonably reflect the “true” metabolism of Lp(a) in subjects who had a predominant apo(a) isoform that contributed substantially to total plasma apo(a) concentration (eg, >70%), or in subjects with 2 apo(a) isoforms of comparable size (eg, differing ≤8 KIV repeats). We identified 9 of the 63 subjects who did not meet the above criteria (ie, a predominant apo(a) isoform with concentration >70% and to the difference in KIV repeats ≤8 KIV repeats). However, removal of these 9 subjects from statistical analyses did not alter the principal findings of the study, implying that our results were not significantly confounded by reliance of our kinetic analyses on the predominant apo(a) isoform alone. However, a more detailed study is required to examine the kinetics of 2 different apo(a) isoforms within the same individual.

Measurement of Lp(a)‐apoB‐100 kinetics also could provide additional information for more fully addressing the transport of Lp(a) particles. In a substudy of our cohort,^6^ we previously reported that in 16 individuals with a wide range of apo(a) isoform size (16–32 KIV repeats) the FCRs of Lp(a)‐apo(a) and Lp(a)‐apoB were similar and highly correlated at pre‐ and postintervention within the same individual (*r*=0.982 and 0.995, respectively). This suggests that the metabolism of both protein components of the Lp(a) particle are tightly coupled across a wide spectrum of different apo(a) isoform sizes. These findings were also observed in interventions that alter or do not alter the plasma concentration of Lp(a).^6^ We need to consider that our observations cannot be generalized in that they were made in normolipidemic white men. Our findings may be different in hyperlipidemic individuals (ie, elevated LDL‐cholesterol, Lp(a), and triglycerides) or other pathological conditions (eg, ASCVD, diabetes mellitus, metabolic syndrome) as well as in pre‐/postmenopausal women or nonwhites. Additionally, the study is limited by its cross‐sectional design and notably correlations are not proof of causality.

## Conclusions: Translational Implications

Our kinetic study advances knowledge of the in vivo physiology of Lp(a) particles and identifies the rate of apo(a) production as the principal determinant of apo(a) concentration in the circulation of normolipidemic, white males. That PR determines apo(a) concentration against background statin and evolocumab suggests that therapies for effectively lowering plasma Lp(a) concentration could target the hepatic synthesis and secretion of apo(a). Although we only studied subjects under physiological condition, our findings may extend to subjects with elevated Lp(a) concentration. Our findings support use of apo(a) antisense oligonucleotides or silent RNAs for lowering Lp(a) by targeting apo(a) production.^39^, ^41^ While effectively increasing the clearance of LDL‐apoB‐100 and lowering LDL‐cholesterol,^6^ statins do not reduce plasma Lp(a) concentrations. Hepatic secretion of apo(a) remains a principal target of therapy, despite the fact that proprotein convertase subtilisin‐kexin type 9 inhibitor can lower Lp(a) by altering the kinetics of Lp(a) particles. Whether this approach decreases the incidence of ASCVD in subjects with elevated Lp(a) remains to be demonstrated in clinical outcome studies.

## Sources of Funding

Amgen Inc funded this study.

## Disclosures

Watts has received honoraria for advisory boards and speakers bureau or research grants from Amgen Inc, Sanofi, Regeneron, Kowa, Gemphire, and Genfit. Coll and Wasserman are current employees of Amgen Inc and own Amgen stock/stock options. The remaining authors have no disclosures to report.
